# Effect of postpartum depression on exclusive breast-feeding practices in sub-Saharan Africa countries: a systematic review and meta-analysis

**DOI:** 10.1186/s12884-020-03535-1

**Published:** 2021-02-08

**Authors:** Demelash Woldeyohannes, Yohannes Tekalegn, Biniyam Sahiledengle, Dejene Ermias, Tekele Ejajo, Lillian Mwanri

**Affiliations:** 1 Department of Public Health, College of Medicine and Health Science, Wachemo University, Hossana, Ethiopia; 2 Department of Public Health, College Health Science, Madda Walabu University, Bale Robe, Ethiopia; 3 College of Medicine and Public Health, Flinders University, Health Sciences Building, Sturt Road, Bedford Park, Adelaide, SA 5001 Australia

**Keywords:** Exclusive breast feeding practices, Postpartum depression, Sub-Saharan Africa

## Abstract

**Background:**

Postpartum depression (PPD) is a serious mood disorder that affects behavioural, physical and mental health of women and newborn after childbirth. Although a wide range of research have been conducted on maternal and infant health outcomes, the effect of postpartum depression on exclusive breastfeeding practices remains ambiguous, and needs addressing. The aim of this study was to assess the effect of postpartum depression on exclusive breast feeding practices in sub-Saharan African countries.

**Methods:**

PubMed, Google Scholar, Science Direct and Cochrane Library were systematically searched for relevant articles published between 2001 and 2020. STATA version 14 was used to calculate the pooled odd ratio with 95% confidence intervals (95% CI). The DerSimonian and Laird random effects meta-analysis was used to measure the effect of postpartum depression on exclusive breast feeding practices. The heterogeneity and publication bias were assessed by using I^2^ test statistics and Egger’s test, respectively. This review was reported according to the Preferred Reporting Items for Systematic Reviews and Meta-Analysis.

**Result:**

A total of 1482 published articles and gray literatures were retrieved from different databases. Additional articles were identified from the reference list of identified reports and articles. After assessment of obtained articles, studies not meeting the inclusion criteria were excluded. Twenty six studies involving 30,021 population met the inclusion criteria were included in this review. In sub Saharan Africa the overall estimated level of postpartum depression was 18.6% (95% CI: 13.8, 23.4). This review found that postpartum depression had no significant effect on exclusive breast feeding practices (OR = 0.46, 95% CI: 0.18, 1.14).

**Conclusion:**

In Sub Saharan Africa, the prevalence of postpartum depression was lower than the report of World Health Organization for developing Country in 2020. This review reveled that maternal postpartum depression has no significant effect on exclusive breast feeding practices. Thus, the investigators strongly recommend the researchers to conduct primary studies using strong study design in sub-Saharan Africa.

## Background

Depression is a serious mood disorder that is estimated to affect the health of 350 million people worldwide. It is a common mental health disorder that is estimated to affect 10–15% of all mothers in the postpartum period [1–3]. Postpartum depression is a disorder experienced by women following the childbirth and may arise from a combination of hormonal changes, psychological adjustment to motherhood and fatigue [1].

Studies have found that in the first three months after childbirth, 14.5% of women have an episode of minor or major depression, with nearly 40% of these women having experienced symptoms during pregnancy [4, 5].

Postpartum depression has a considerable burden on partners and close family members, affecting social and leisure activities and posing financial challenges within the family. Additionally, postpartum depression has an adverse effect on the marital relationship [6–8].

There has been significant research in developed countries on the prevalence of postpartum depression and its effect on exclusive breast feeding practices. Meta-analyses of these studies have identified past history of psychopathology, emotional disturbance during pregnancy, difficult marital relationships, inadequate social support and stressful life events as the primary risk factors for developing postnatal depression [7, 9, 10].

Mothers with postpartum depression are more likely to have an unhealthy lifestyle, including poor diet and sleep patterns, compared to mothers free of postnatal depression [11–13]. Women with postnatal depression tend to stop breastfeeding earlier than non-depressed mothers [14–16].

Despite some evidence of a higher risk for depression, most low-income and ethnic minority women remain undiagnosed or untreated for postpartum depression [17]. Postpartum depression is a significant public health concern with wide-ranging negative consequences for women and their children [18–20].

Postpartum depression is associated with impairment of the mother-infant bond, which can result in longer-term disruption of the emotional and cognitive development of the infant [7]. Mothers with postpartum depression are less likely to appropriately interpret and respond to infant signals; have more negative than positive emotions toward their infants and are more likely to intrusive in their interactions with their infants [21]. In comparison, a study conducted in South Africa showed no clear effects of postnatal depression on exclusive breast feeding practices, although postnatal depression at two months was found to be associated with low infant weight at 18 months [22].

A study conducted in Malawi found a significant association of postpartum depression with exclusive breast feeding practices. All of the studies conducted in South Africa, Malawi and Ethiopia identified a high prevalence of postpartum depression (34, 26 and 33%, respectively) but failed to identify significant effect of postpartum depression on exclusive breast feeding practices [22–24].

In Ethiopia, postpartum depression was not associated with adverse exclusive breast feeding in any aspects [25]. Such conflicting results from primary studies, coupled with the absence of any systematic reviews focused on the prevalence of PPD and its effect on exclusive breast feeding practices in sub-Saharan African countries, indicate the need for clarification of the effects of maternal postnatal depression on exclusive breast feeding practices. A preliminary search for systematic reviews on this topic was performed in PubMed, CINAHL, DARE and PROSPERO and not found existing systematic reviews that reported the effect of postpartum depression on exclusive breast feeding practices in sub-Saharan African countries. This study seeks to systematically review the best available evidence regarding the effect of postnatal depression on exclusive breast feeding practices in sub-Saharan African countries.

## Method

### Search strategy

A three-step search strategy was used in this review. Firstly, an initial limited search of MEDLINE/PubMed was undertaken, followed by an analysis of the text words contained in the title and abstract and the index terms used to describe the articles. A second search using all identified keywords and index terms was undertaken across databases, including PubMed, Google Scholar, Science Direct, Proquest MedNar and Cochrane Library. Thirdly, the reference list of all identified reports and articles was searched for additional studies.

The initial keywords were postnatal, postpartum, depression, mental disorders and exclusive breast feeding practices. These keywords were used separately and/or in combination using Boolean operators such as “OR” or “AND”. “Depression, postpartum”[All fields] AND (“africa south of the sahara”[MeSH Terms] OR and “prevalence” [Subheading] OR “(“africa south of the sahara”[MeSH Terms]” [All fields] AND “Ethiopia” [MeSH Terms] OR “africa south of the sahara”[All Fields]), “Infant breastfeeding”.

### Study selection

#### Inclusion criteria

**Study setting**: Sub-Sahara Africa.

**Study participants:** Exclusive Breast feeding mothers.

**Publication condition:** All published articles and gray literatures.

**Language:** English language.

**Types of studies:** Observational study designs.

**Publication date:** From January 2001 to June 28, 2020, due to the lack of studies from sub Saharan Africa on the topic before 2001.

**Exclusion criteria:** Unable to access full-texts after two email contacts of the principal investigator.

### Types of exposure

This review considered studies that examined exclusive breast feeding practices in women with postpartum depression versus women without postpartum depression.

Postpartum depression is defined as depression that starts within one month after childbirth and whose symptoms last more than two weeks. We can measure postnatal depression by using the Diagnostic and Statistical Manual of Mental Disorders or Edinburgh Postnatal Depression Scale and self-reporting questionnaire-20. This review excluded studies that have been conducted on mothers with preexisting psychological disorders.

### Outcome of interests

The outcome interest of this review was the pooled prevalence of PPD and its effect on exclusive breast feeding practices in Sub Saharan Africa. Effect size was estimated in the form of log odds ratios.

### Types of studies

This review considered studies with observational study designs: prospective, retrospective follow up studies and cross-sectional studies reporting an association between postpartum depression and exclusive breast feeding practices were considered for this review.

### Methodological quality

Quality of each original study was assessed independently (DW, YT) by using Newcastle-Ottawa Scale (NOS) quality assessment tool [26]. Any disagreements were resolved by taking the mean score. Finally, studies with a scale of ≥5 out of 10 were considered as achieving high quality. Any disagreements that arise between the reviewers were resolved through discussion with a third reviewer (BS) (Table 1).

**Table 1:** Characteristics of studies included in the systematic review and meta-analysis on the effect of postpartum on exclusive breast feeding practices in Sub-Saharan Africa, 2020

Author	Year	Country	Sub regions	Study setting	Study design	Sample size	Prevalence of PPD with CI	Tool Used
Adamu and Adinew [27]	2018	Ethiopia	Eastern Africa	Facility Based	Cross-sectional study	618	23.3 (19.9, 26.6)	EPHD
Anokye et al. [28]	2018	Ghana	Western Africa	Facility Based	Cross-sectional study	257	7 (3.9, 10.1)	PHQ
Azale et al. [29]	2016	Ethiopia	Eastern Africa	Community Based	Cross-sectional study	3147	11.2 (10.1, 12.3)	PHQ
Bitew et al. [30]	2019	Ethiopia	Eastern Africa	Facility Based	Prospective study	1240	22.1 (19.8, 24.4)	PHQ
Christodoulou et al. [31]	2019	South Africa	Southern Africa	Facility Based	Prospective study	468	15.6 (12.3, 18.9)	EPHD
Dow et al. [32]	2014	Malawian	Southern Africa	Community Based	Prospective study	492	10.9 (8.2, 13.7)	EPHD
Garman et al. [33]	2019	South Africa	Southern Africa	Facility Based	Prospective study	351	9.4 (6.3, 12.5)	HDRS
Huang et al. [34]	2018	Uganda	Eastern Africa	Community Based	Cross-sectional study	303	28.1 (22.9, 33.1)	PHQ
Kerie et al. [35]	2018	Ethiopia	Eastern Africa	Facility Based	Cross-sectional study	408	33.8 (29.2, 38.4)	EPHD
Khalifa et al. [36]	2015	Sudan	Eastern Africa	Facility Based	Cross-sectional study	236	9.3 (5.6, 13.1)	EPHD
Khalifa et al. [37]	2018	Sudan	Eastern Africa	Facility Based	Cross-sectional study	223	3.6 (1.1, 6.1)	EPHD
Madeghe et al. [38]	2016	Kenya	Eastern Africa	Facility Based	Cross-sectional study	200	13 (8.3, 17.7)	EPHD
Juliet E.M Nakku [39]	2006	Uganda	Eastern Africa	Facility Based	Cross-sectional study	465	8.2 (5.7, 10.7)	SRQ
R. Ndokera and C. MacArthur [40]	2010	Zambia	Southern Africa	Facility Based	Cross-sectional study	278	9.7 (6.2, 13.2)	SRQ
Nhiwatiwa [41]	1998	Zimbabwe	Southern Africa	Community Based	Prospective study	500	19 (15.6, 22.4)	SSQ
Ongeri et al. [42]	2018	Kenya	Eastern Africa	Facility Based	Prospective study	171	18.7 (12.9, 24.6)	EPHD
Osok et al. [43]	2018	Kenya	Eastern Africa	Facility Based	Cross-sectional study	176	32.9 (26, 39.9)	PHQ
P.G. Ramchandani et al. [44]	2009	South Africa	Southern Africa	Facility Based	Cross-sectional study	1035	16.4 (14.2, 18.7)	
S. Shamu et al. [45]	2016	Zimbabwe	Southern Africa	Facility Based	Cross-sectional study	824	21.8 (19, 24.7)	SQR
Stellenberg EL, [46]	2015	South Africa	Southern Africa	Community Based	Cross-sectional study	159	50.3 (42.5, 58.1)	EPHD
Robert C. Stewart [47]	2010	Malawi	Western Africa	Facility Based	Cross-sectional study	501	30.5 (26.5, 34.5)	SQR
Toru et al. [48]	2018	Ethiopia	Eastern Africa	Community Based	Cross-sectional study	456	22.4 (18.5, 26.2)	CES-D
Wemakor and Iddrisu [49]	2018	Ghana	Western Africa	Community Based	Cross-sectional study	200	33.5 (26.9, 40)	CES-D
Weobong et al. [50]	2015	Ghana	Western Africa	Facility Based	Prospective study	13,360	3.4 (3.1, 3.7)	SQR
Rohde SS [51].	2014	South Africa	Southern Africa	Facility Based	Cross sectional study	3494	15.9 (14.8, 17.2)	EPHD
Abdulai, H [52].	2019	Ghana	Western Africa	Facility Based	Cross sectional study	300	27.3 (22.3, 32.4)	SHQ

Note: *CES-D* Centre for Epidemiologic Studies-Depression, *EPHD* Edinburgh Postnatal Depression, *PHQ* Patient Health Questionnaire, *SRQ* Self-reporting Questionnaire, *SSQ* Shona Symptom Questionnaire

### Data extraction

The standard data extraction tool was prepared in a Microsoft Excel spreadsheet. This included the specific details about the exposures, populations, study methods and outcomes of significance to the review question and specific objectives. The authors of primary studies were contacted by email in case there is incomplete information. Two reviewers (DW and YT) extracted the data independently. Any differences among reviewers were negotiated with review team members until agreement was reached.

### Data synthesis

Data were analyzed using STATA version 14. Heterogeneity was assessed using the standard *I*^*2*^ and visual inspection of forest plots. To check publication bias, both objective and subjective (funnel plot) methods were used. Mainly, objective methods such as Eggers’ and Beggs’ tests (*p*-value < 0.05) were used to assess publication bias [53, 54]. The result of Eggers’ test revealed statistically significant publication bias (*p*-value < 0.001). Finally, Duval and Tweedie’s nonparametric trim and fill analysis was performed to account for this publication bias. The pooled prevalence and effect size was expressed as standardized mean differences and their 95% confidence intervals were calculated using the DerSimonian and Laird method. Moreover, odds ratios and their 95% confidence intervals were calculated.

## Result

### Characteristics of the studies

**Fig. 1:**
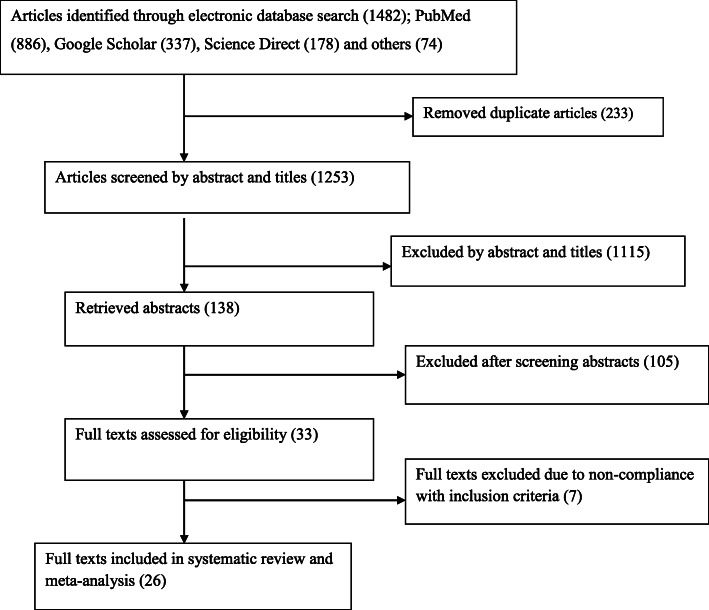
Flow diagram shows the study selection of the meta-analysis of the effect of postpartum depression on exclusive breast feeding practices in Sub-Saharan Africa, 2020

A total of 1482 studies were retrieved from different databases. After removing the duplicate articles (*n* = 233) and after reading titles and abstracts, 1115 articles were further excluded due not in line with our inclusion criteria. After reading the abstracts, additional 105 articles were excluded. Finally, a total of 33 full text studies were downloaded and assessed their eligibility. Among full text articles, seven articles were excluded because they did not meet inclusion criteria (in four papers outcome of interests was not reported and the three of papers were no a primary study). Finally, 26 articles were used for the meta-analysis. This process has been reported (Fig. 1) according to the preferred reporting items for systematic reviews and meta-analyses [55]. Of the included articles: 19 were cross-sectional study designs [27–29, 34–40, 43–49, 51, 52] and seven studies were a prospective cohort studies [30–33, 41, 42, 50] and they comprised a population of 30,021. The minimum sample size from the included study was 159 [46] from South Africa, whereas the maximum sample size from the included study was 13,360; from Ghana [50]. The prevalence of postpartum depression in Sub-Saharan Africa ranged from 3.4% in Kintampo Health Research Centre in Ghana [50] to 50.3% in rural South Africa [46]. The summary of characteristics of included studies is reported in Table 1 below.

#### Pooled prevalence of postpartum depression

**Fig. 2:**
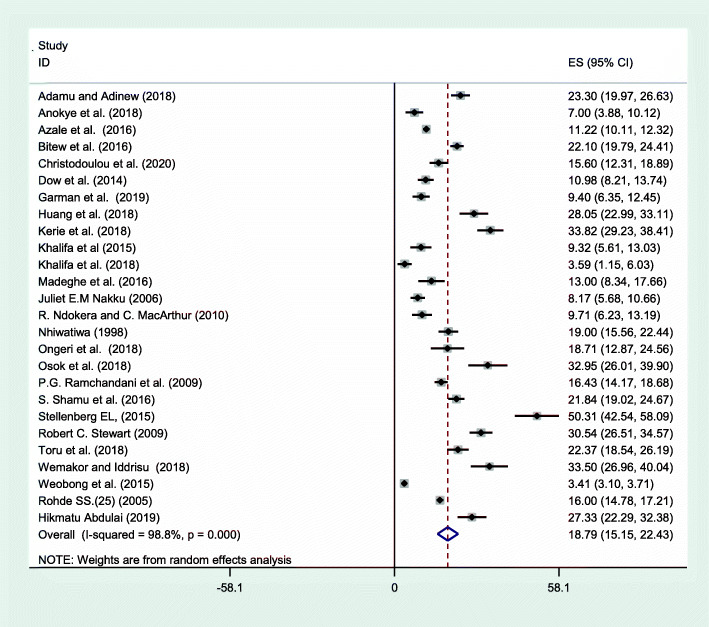
Forest plot displaying the pooled prevalence of postpartum depression in Sub-Saharan Africa, 2020

**Fig. 3:**
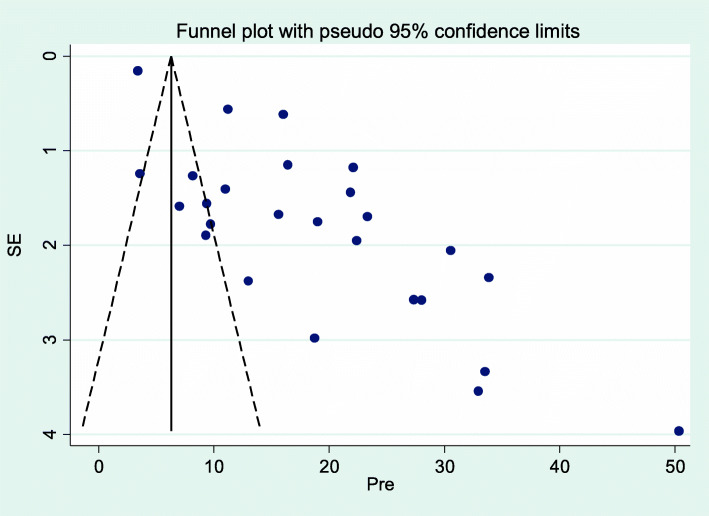
Funnel plot of pool prevalence of postpartum depression in Sub-Saharan Africa, 2020

Overall random effects estimate of the postpartum depression across Sub Saharan Africa studies was 18.79% (95% CI: 15.15, 22.43%) (Fig. 2). This observed effect size varies somewhat from study to study. Test statistics results showed high heterogeneity (*I*^*2*^ = 98.8%, *p* < 0.001) and Eggers’ test (*p*-value < 0.001) revealed significant publication bias. The possible publication biases were visualized thru funnel plots. Symmetrical large inverted funnels resembled the presence of publication biases (Fig. 3). After we applied trim and fill meta-analysis, the overall random effect estimates of postpartum depression across studies was 18.92% (95% CI: 15.1, 22.7%) Table 2. The prevalence of postpartum depression vary in sub regions of Sub Saharan Africa, which was 20.2% (95% CI: 7.7, 32.6) in western Africa, 18.6% (95% CI: 13.8, 23.4) in Eastern Africa and 18.3% (95% CI: 13.4, 23.3) in southern Africa (Table 3).

**Table 2:** Results from the trim-and-fill method for publication bias in 26 studies on effect of postpartum depression on exclusive breast feeding practices in Sub Saharan Africa, 2020

Method	Pooled Estimate (%)	95% CI		Z value	*p*- values	Estimated Number of studies
		Lower	Upper			
Fixed effect	6.293	6.027	6.560	46.258	0.001	26
Random	18.791	15.154	22.428	10.125	0.001

**Table 3:** Subgroup level of postpartum depression by sub regions of Sub Saharan Africa, 2020

Variable	Characteristics	Included studies	Sample size	Estimated prevalence of PPD (95% CI)
Sub region	Eastern Africa	12	7643	18.6 (13.8, 23.4)
	Western Africa	5	14,618	20.2 (7.7, 32.6)
	Southern Africa	9	7601	17.7 (14.0, 21.5)

#### Effects of postpartum depression on exclusive breast feeding practices

**Fig. 4:**
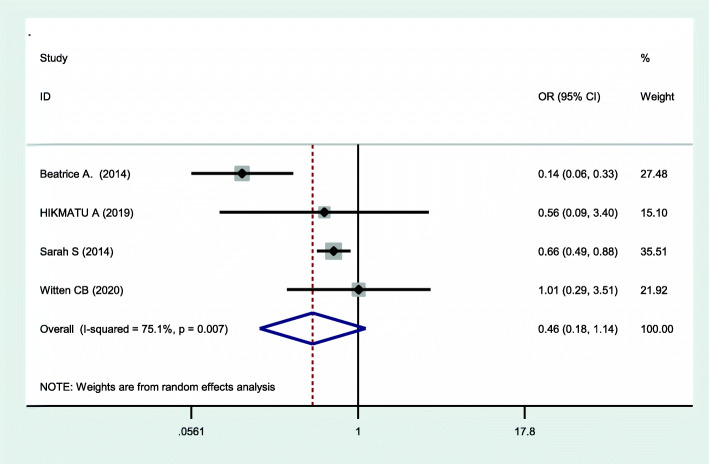
Forest plot of the pooled effect of postpartum depression on exclusive breast feeding practices in Sub-Saharan Africa, 2020

**Fig. 5:**
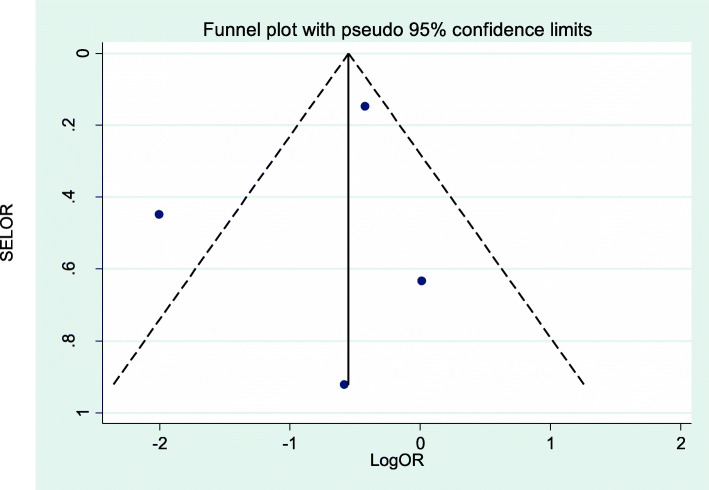
Funnel plot of the effect of postpartum depression on exclusive breast feeding practices in Sub-Saharan Africa, 2020

This systematic review and meta-analysis assessed the effect of postpartum depression on exclusive breast feeding practices. Four studies assessed the effect of effect of postpartum depression on the exclusive breast feeding practices. Heterogeneity was moderate across studies (I^2^ = 75.1%, *p = 0.007*), which enabled us to use a random effects model. Using this method, our meta-analysis found that postpartum depression had no significant effect on exclusive breast feeding practices (OR = 0.46, 95% CI: 0.18, 1.14) (Fig. 4). We explored possible sources of heterogeneity using Univariate meta-regression using publication year and sample size as covariates. However, none of these variables were statistically significant for explaining heterogeneity (Table 4). The presence of publication bias was also assessed using funnel plots and Eggers’ and Beggs’ statistical tests at the 5% significance level. The funnel plot shows a symmetrical distribution (Fig. 5). Egger and Beggs’ tests showed no significant publication bias with *p*-values of 0.642 and 0.734, respectively. The influence of a single study on the overall meta-analysis estimate was assessed by sensitivity analysis using a random effects model and revealed that no single study influenced the overall effect of postpartum depression on exclusive breast feeding practices.

**Table 4:** Related factors with heterogeneity of the effects of postpartum depression on exclusive breast feeding practices in Sub-Saharan Africa, 2020

Variable	Coefficients	***P***-value
Year	0.2650735	0.533
Sample size	−0.0013525	0.103

## Discussion

By 2020, the World Health Organization projected that depression will become the second and significant predictor of the global burden of disease [56]. One in five women in low- and middle-income countries developed postpartum depression according to reviews conducted in Low and Middle Income Countries (LMICS) and a review conducted in Africa [57, 58]. Evidence has shown that common mental disorders, including depression, during the postpartum period are more prevalent compared with non-pregnancy periods [59].

A study conducted in Malawi found the effect of postpartum depression on exclusive breast feeding practices. All of the studies conducted in South Africa, Malawi and Ethiopia identified a high prevalence of postpartum depression (34, 26 and 33%, respectively) but failed to identify the effect of postpartum depression on exclusive breast feeding practices [24, 25, 60]. Whereas, in this review the prevalence of postpartum depression in Sub Saharan Africa was 18.9% this finding was higher than a review conducted in Africa [61]. The prevalence of postpartum depression varies in sub regions of Sub Saharan Africa, which was 20.2% in western Africa, 18.6% in Eastern Africa and 18.3% in southern Africa. This showed that there is conflicting result seen for the prevalence of postpartum depression and its effect on exclusive breast feeding practices [18, 19]. Hence, this systematic review and meta-analysis is perhaps the first of its kind to be conducted in Sub-Saharan Africa to examine the effect of postpartum depression on exclusive breast feeding practices.

This systematic review and meta-analysis revealed that postpartum depression has no significant effect on exclusive breast feeding practices. This finding is in agreement with individual studies conducted in the Republic of Korea [62], South Africa [60], Malawi [24], Ethiopia [25], Malaysia [63], Brazil [64], and Saudi Arabia [36], whereas the finding of this study was in contrast to a qualitative systematic review [65], a study conducted in Saudi Arabia [66] and a study conducted in Mexico [66]. These disagreements could be the result of sociodemographic and socioeconomic differences between the countries. The other potential explanations for the observed differences might be the use of different tools to assess postpartum depression. The difference in sample size and different study periods may be the additional causes for these variations. In the above studies, the results were based on individual studies, but this systematic review and meta-analysis pooled the effect of postpartum depression on exclusive breast feeding practices based on the four studies conducted in different study areas and periods. However, a study conducted in Canada showed that the effect of postpartum depression on infant feeding depends on the duration of exclusive breast feeding [15].

### Limitations of the study

Maximum efforts have been made to include all published articles and gray literatures from sub-Saharan African countries. Moreover, 97% of the studies in this meta-analysis were cross-sectional study design. Chicken egg relationships, therefore, cannot be shown in this review.

## Conclusions

In Sub Saharan Africa, the prevalence of postpartum depression was lower than the report of WHO for developing Country in 2020. This review revealed that maternal postpartum depression has no significant effect on exclusive breast feeding practices. Thus, the investigators strongly recommend the researchers to conduct primary studies with strong study design that can rule out the effect of PPD on breast feeding practices in sub-Saharan Africa.

## Data Availability

This study was based on a literature review of published studies in Sub-Saharan Africa. Anyone who needs to access the data can contact the author concerning the studies included in the analysis. The reference list can also be used to directly access the articles.

## Abbreviations

AOR: Adjusted odds ratio
PPD: Postpartum depression
PRISMA: preferred reporting items for systematic reviews and meta-analyses
